# Analysis of pooled genome sequences from Djallonke and Sahelian sheep of Ghana reveals co-localisation of regions of reduced heterozygosity with candidate genes for disease resistance and adaptation to a tropical environment

**DOI:** 10.1186/s12864-019-6198-8

**Published:** 2019-11-07

**Authors:** M. Yaro, K. A. Munyard, E. Morgan, R. J. N. Allcock, M. J. Stear, D. M. Groth

**Affiliations:** 1 School of Biomedical Sciences, CHIRI Biosciences Research Precinct, Faculty of Health Sciences, Curtin University, GPO Box U1987, Perth, WA 6845 Australia; 2 The University of Western Australia, 35 Stirling Highway, Crawley, Perth, WA Australia; 3 Pathwest Laboratory Medicine WA, QEII Medical Centre, Monash Avenue, Nedlands, 6009 Australia; 4 Agribio centre for Agribioscience, La Trobe University, Melbourne, Australia; 5 Institute of Biodiversity, Animal Health and Comparative Medicine University of Glasgow, Bearsden Road, Glasgow, G61 1QH UK

**Keywords:** Djallonke, Sahelian, Heterozygosity, Disease resistance, Trypanotolerance, Nematode, Adaptation, Sheep, Africa

## Abstract

**Background:**

The Djallonke sheep is well adapted to harsh environmental conditions, and is relatively resistant to Haemonchosis and resilient to animal trypanosomiasis. The larger Sahelian sheep, which cohabit the same region, is less well adapted to these disease challenges. Haemonchosis and Trypanosomiasis collectively cost the worldwide animal industry billions of dollars in production losses annually.

**Results:**

Here, we separately sequenced and then pooled according to breed the genomes from five unrelated individuals from each of the Djallonke and Sahelian sheep breeds (sourced from Ghana), at greater than 22-fold combined coverage for each breed. A total of approximately 404 million (97%) and 343 million (97%) sequence reads from the Djallonke and Sahelian breeds respectively, were successfully mapped to the sheep reference genome Oar v3.1. We identified approximately 11.1 million and 10.9 million single nucleotide polymorphisms (SNPs) in the Djallonke and Sahelian breeds, with approximately 15 and 16% respectively of these not previously reported in sheep. Multiple regions of reduced heterozygosity were also found; 70 co-localised within genomic regions harbouring genes that mediate disease resistance, immune response and adaptation in sheep or cattle. Thirty- three of the regions of reduced heterozygosity co-localised with previously reported genes for resistance to haemonchosis and trypanosomiasis.

**Conclusions:**

Our analyses suggest that these regions of reduced heterozygosity may be signatures of selection for these economically important diseases.

## Background

The Djallonke sheep is recognised for its natural ability to withstand a harsh, hot and humid tropical climate, where it is faced with the challenges of persistent drought, diseases and feed scarcity [1, 2]. Adaptation is probably a consequence of natural selection over several millennia [3–5]. Genomic regions adjacent to loci under adaptive selection over time are usually characterised by low heterozygosity [6]. The most important livestock diseases are trypanosomiasis and haemonchosis [7–10]. The natural ability of the Djallonke to survive and remain productive under trypanosome challenge with very low mortality and without the aid of trypanocidal drugs is referred to as trypanotolerance [11]. Trypanosomiasis in sub Saharan Africa is estimated to cause annual losses of more than 4.5 billion dollars (US$) through direct and indirect production costs [12, 13]. Development of trypanotolerance is considered to be the most economical and sustainable option for combating African trypanosomiasis [9, 14, 15]. The potential of this trypanotolerant trait in mitigating the disease in Africa has recently been reviewed [16]. However, because Djallonke sheep have a relatively small mature body weight (between 20 kg to 30 kg [17];) farmers often cross-breed them with the larger, but more disease susceptible, Sahelian breed.

In spite of the importance of the Djallonke and Sahelian sheep to the region, genetic studies are scarce. There are no records of whole genome variant characterisation in either the Djallonke or Sahelian breeds, besides our preliminary report at the International society of animal genetics conference [18]. The objectives of this study are: i) to identify and document variants in each breed and ii) to use these to investigate putative candidate genetic regions in both breeds.

## Methods

### Animals

Four ewes and one ram of the Djallonke breed (DJ) were selected from the National Open Nucleus Breeding Station (ONBS) dedicated for Djallonke Sheep in Ejura in the Ashanti region (longitude 01^o^ 28′W and latitude 06^o^ 41′ N). Five Sahelian (SA) ewes were selected from the National Sheep Breeding Station in Pong Tamale (longitude 00^o^ 54′W and latitude 09^o^ 38′ N). All sheep were reproductively mature (13–24 months old) and were chosen in consultation with the management of the breeding stations to represent unrelated animals that were true to breed type (phenotypically similar to the breed ideal). The management relied on stock records for determination of relatedness among sheep on two breeding stations. Approximately 9 ml of blood was collected via the jugular vein into disodium EDTA vacutainers. All sampled sheep were monitored on farm for at least 24 h post sampling and no adverse effect was recorded. No sheep were sacrificed during this study. The samples were transported at 0^o^ – 4^o^ C to the laboratory, centrifuged at 800 x g for 3 min at room temperature (15-25 °C) with the rotor bucket brake off. The buffy coat was used immediately for genomic DNA extraction or was stored at -20 °C. Genomic DNA was extracted from each of the buffy coat samples using the Zymo Quick-gDNA™ MiniPrep DNA purification Kit (according to the manufacturer’s protocol). DNA quality and concentration were assessed using agarose gel electrophoresis (1% in 1xTAE) and by Nanodrop spectrophotometry.

### Library construction and sequencing

For each individual, 100 ng of DNA was sheared using the Covaris S2 System, to generate a broad range of DNA fragments with sizes from 100 to 1000 bp. The DNA fragments were ligated to T-overhang adaptors with the NEB Next Ultra kit (New England Biosciences). Each animal had a unique barcode (Ion Xpress Barcodes, Life Technologies). Fragments of approximately 300-330 bp were size-selected using the E-gel system (Invitrogen), and recovered fragments were further purified using AMPure XP SPRI beads (Beckman). Equimolar amounts of each library were combined and amplified using an Ion Chef system (ThermoFisher Scientific) via emulsion PCR, then sequenced on an Ion Proton™ system (ThermoFisher Scientific) using a PI chip. Genomic DNA from the ten individuals was separately sequenced, and the sequencing reads were then pooled by breed. After filtering and trimming, an average of 10 and 13% of the reads were excluded due to low quality, and 26 and 28% excluded due to polyclonality for the Djallonke and Sahelian samples, respectively. Coverage analysis was performed on a total (post QC) of 73 Gbp of sequenced data, comprising 404,755,012 pooled reads (average read length 185.4 nucleotides) and 57.6 Gbp comprising 303,136,043 pooled reads (average read length 176.6 nucleotides) for Djallonke and Sahelian sheep, respectively. The genome coverage depth obtained was calculated as being 27.90x and 22.01x for the Djallonke and the Sahelian respectively, and covered 97% of sheep reference assembly v3.1. All the variants were submitted to the European variant archive of the European Bioinformatics Institute with the accession number PRJEB15642.

### Mapping and pre-processing of reads

Base calling, de-multiplexing, quality control (QC) and alignment pre-processing [19] was completed using Torrent Suite 4.6 on a Torrent Server (ThermoFisher Scientific). Briefly, polyclonal and uniformly low-quality reads were removed, and the remaining reads were trimmed from the 3′ end only. Mapping was also performed within Torrent Suite 4.6, using the Torrent mapping alignment program (TMAP). Individual libraries were mapped to the sheep reference genome Oar v3.1 (University of California, Santa Cruz (UCSC)). For each of the sheep breeds, all individual BAM files were merged and sorted using SAMtools v0.1.19-44,428 cd [20], and coverage analysis was performed for both the individual and combined datasets through automated plugins in TorrentSuite 4.6. Duplicate reads were removed using Picard Tools v1.122.

### Variant calling pipeline

Genome Analysis Tool Kit version 3.2.2 (GATK) RealignerTargetCreator and IndelRealigner were used to produce realignments of the pooled BAM files for each breed. GATK HaplotypeCaller was used in GVCF mode to call intermediate genome-wide variants separately for the pooled DJ genomes and pooled SA genomes, producing two pooled genomic variant call format (gvcf) files. GATK GenotypeGVCFs was then used to perform a joint genotyping of the two pooled gvcf files with minimum standard confidence thresholds for both calling and emitting variants set at 30 to produce a composite pooled variant call format (vcf) file (Pooled-Sheep VCF). This analysis was selected to ensure good quality variant calling and reduce false discovery rates. Finally, VCFtools (v0.1.15) [21] was used to extract individual Djallonke and Sahelian samples from the composite joint genotyped vcf into separate vcf files, which were used for downstream analyses.

### Genetic relationship matrix

To determine genetic relatedness, a genomic relationship matrix (GRM) was computed on a composite vcf file that contained all the 10 vcf files generated from both the Djallonke and the Sahelian samples, using the Genome-wide Complex Trait Analysis (GCTA) software [22, 23]. Furthermore, Principal Components analysis (PCA) was used to compare the autosomal genomes of all individual samples to determine population substructure for each breed [24], and assess the genetic relatedness using the SNPRelate implemented in R CRAN (http://cran.r-project.org) [25].

### Detection of regions of reduced heterozygosity

HomSI (Homozygosity Stretch Identifier) was used to identify regions of reduced heterozygosity in both genomes [26]. The Djallonke genome was designated as the index case and compared against the Sahelian as the unaffected case for input settings for the HomSI analysis. Analysis of runs of homozygosity in Djallonke and Sahelian using HomSI, with the stringent settings of 5 Mb window size and 10 kb sliding size, allowed the capturing of a wide spectrum of different lengths of homozygosity throughout the genome [26–29]. Integrative Genomics Viewer (IGV 2.3.46, www.BroadInstitute.org) was used to view vcf and BAM file tracks aligned to the sheep reference genome Oar v3.1, selecting regions based on genomic coordinates of regions of contrasting reduced heterozygosity identified by HomSI in order to identify candidate genes. Prominent regions were investigated using IGV for the specific genes of interest [30, 31] and were used to identify candidate genes within the region. For every prominent region of low heterozygosity in the HomSI output, the co-localised candidate gene or genes were inferred from the available Ensembl (Release 85 and 86) annotated sheep reference assembly (version 3.1) or from the conserved synteny for other mammalian genomes from the Ensembl genome database [32–34]. The approach presented here for investigating genetic evidence of trypanotolerance and resistance to haemonchosis was by directly linking regions with contrasting heterozygosity in this dataset to the reported candidate genes in the database for Animal Quantitative Trait Loci (Animal QTLdb) [35] and other previously published genetic association studies for the two traits. There have been several previous genomic investigations of resistance to nematode infection including *H. contortus* in multiple sheep breeds and these results were compared with our Djallonke and Sahelian sheep results [36–40]. In contrast, there has been no previous genomic investigation of trypanosomiasis in any sheep breed; therefore, comparison was made with the reported trypanotolerance associated candidate genes in Ndama cattle [41–45].

### Annotation and functional analysis of genomic variants

As there were unequal numbers of males and females used between the two breeds, for the purpose of a balanced comparison, autosomal chromosomes were extracted from each of the pooled vcf files using VCF tools v0.1.15. Known SNPS were annotated in the vcf files with SnpSift v4.2 (annotate command) using the Ensembl Release-85 Variation reference vcf for *Ovis aries* as the database. (ftp.ensembl.org/pub/release-85/variation/vcf/ovis_aries/) [32]**.** SnpEff v4**.**2 (Cingolani et al., 2012) was used for functional annotation of identified autosomal SNPs in both the Djallonke and Sahelian genomes based on the Ensembl sheep genome assembly Oar_v3.1.82. Pairwise comparison of Genomic SNPs and INDELS for Sahelian and Djallonke sheep was computed using the BEDTools suite v. 2.26.0 [46].

## Results

### Genetic relationship matrix and principal component analysis

**Fig. 1:**
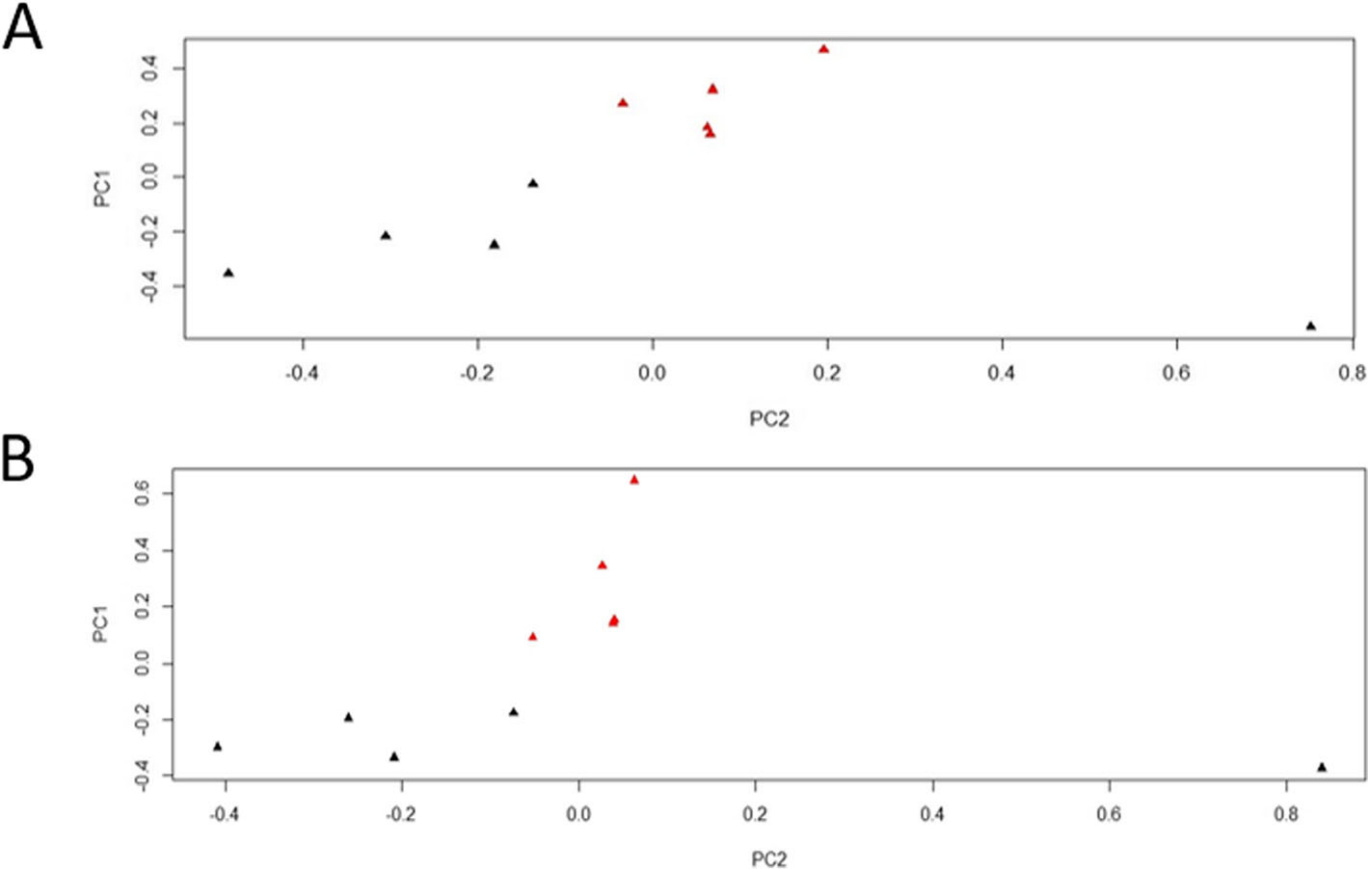
PCA of Autosomal Genomes for 5 Djallonke (DJ) and 5 Sahelian (SA) sheep showing distinct clustering of the two breeds

The GRM computed for these datasets supports the assumption that the ten individual animals sampled were unrelated. Additional file 1shows the GRM output for this analysis. The PCA computed for the 10 individual datasets from the two sheep breeds showed distinct clustering for the two breeds (Fig. 1).

### Comparison of genomes

On average, there was one variant every 191 base pairs (bp) in the Djallonke sheep and 1 variant every 193 bp in the Sahelian sheep (Table 2). Transition to transversion ratios were similar in the Djallonke (2.47) and Sahelian (2.48) sheep (Table 1). The estimated missense to silent SNP ratios were also similar between the Djallonke and Sahelian sheep (0.69 and 0.68 respectively). Similarly, the two datasets had equal insertion to deletion ratios (0.38). Similar proportions of SNPs, insertions and deletions were observed for Djallonke (86%: 4%: 10%) and Sahelian (87%: 4%: 9%) sheep. Approximately 84% of variants in both Djallonke and Sahelian sheep were present in the Ensembl Variation database (release 85) hence approximately 16% are unidentified variants (Table 1). Analysis of only SNPs, however, revealed that approximately 94% were present in the database (Table 1), indicating that approximately 6% of SNPs were previously unreported in sheep.

**Table 1:** Summary of SnpEff variant analysis of Djallonke and Sahelian sheep genomes

	Djallonke all variants	Sahelian all variants	Djallonke SNP only	Sahelian SNP only
Number of variants	12,821,836	12,654,761	11,100,619	10,978,689
Number of known variants	10,764,740 (83.956%)	10,671,465 (84.328%)	10,460,303 (94.232%)	10,372,765 (94.481%)
Number of multi-allelic entries	9235	9199	5546	5534
Number of effects	14,652,590	14,454,278	12,641,434	12,496,971
Variant rate	1 per 191 bases	1 per 193 bases	1 per 220 bases	1 per 223 bases
Ts/Tv ratio	2.4736	2.4819	2.4736	2.4819

### The distribution of variants by chromosome

The distribution of the variants (i.e. the sum of SNPs, insertions and deletions) was similar across all the autosomes, with chromosomes 11 and 26 having the lowest and highest frequencies for variants in both breed genomes (Table 2), respectively. A total of 12,821,836 and 12,654,761 variants were identified in Djallonke and Sahelian sheep, respectively, with 12,556,638 (96.30%) shared between the two genomes. In total, 324,760 (2.49%) variants were specific to the Djallonke breed, whereas 158,085 (1.21%) variants were specific to the Sahelian breed. Therefore, the total number of variants identified for the two breeds is 13,039,483. Analysis with BEDTools intersect indicated that 242,572 SNPs and 82,609 indels were specific to the Djallonke, whereas 120,652 SNPs and 37,762 indels were unique to the Sahelian breed.

**Table 2:** Comparison of the Djallonke and the Sahelian Genomes

Chromosome	Length (bases)	Djallonke		Sahelian	
		Variants	Ratio of nucleotides to variants	Variants	Ratio of nucleotides to variants
1	275,612,895	1,415,883	194	1,397,799	197
2	248,993,846	1,252,169	198	1,236,471	201
3	224,283,230	1,126,647	199	1,112,239	201
4	119,255,633	614,660	194	606,652	196
5	107,901,688	547,117	197	540,304	199
6	117,031,472	638,104	183	629,707	185
7	100,079,507	521,899	191	514,989	194
8	90,695,168	463,424	195	457,602	198
9	94,726,778	508,149	186	501,452	188
10	86,447,213	458,020	188	451,926	191
11	62,248,096	309,854	200	305,905	203
12	79,100,223	418,404	189	412,899	191
13	83,079,144	420,072	197	414,577	200
14	62,722,625	313,626	199	309,445	202
15	80,923,592	431,999	187	426,246	189
16	71,719,816	397,765	180	392,498	182
17	72,286,588	390,027	185	384,875	187
18	68,604,602	361, 551	189	356,643	192
19	60,464,314	314,693	192	310,777	194
20	51,176,841	272,206	188	268,369	190
21	50,073,674	279,334	179	275,226	181
22	50,832,532	279,610	181	275,533	184
23	62,330,649	342,228	182	337,640	184
24	42,034,648	233,669	179	230,626	182
25	45,367,442	259,240	175	256,130	177
26	44,077,779	251,486	175	248,131	177
Total	2,452,069,995	12,821,836	191	12,654,761	193

### Distribution of autosomal SNPs by genomic region

Most SNPs in both sheep breeds were intergenic or intronic (Table 3), with approximately 1% located in the remaining genic regions (i.e. untranslated regions (UTR), exons, and splice sites). Although the Djallonke sheep had a higher number of SNPs than the Sahelian sheep, the ratios of the SNPs in these three regions (intergenic, intronic and “other” genic regions including exons) were similar for Djallonke (68.78%: 30.04%: 1.18%) and Sahelian sheep (68.81%: 30.02%: 1.17%). A comparison of the “other” genic category revealed similar proportions of synonymous, non-synonymous, splice site, UTR and miscellaneous variants for each breed (Table 3).

**Table 3:** Comparison of Autosomal SNPs by type and functional category

Variant types	Djallonke	Sahelian
Functional class		
Missense	33,984 (40.7%)	33,395 (40.5%)
Nonsense	344 (0.41%)	338 (0.41%)
Silent	49,171 (58.9%)	48,732 (59.1%)
Intergenic region	8,861,516	8,747,769
Upstream gene	611,224	601,689
Downstream gene	605,975	596,609
Intronic	4,413,052	4,350,066
Intragenic	199	181
3 prime UTR	29,548	28,988
5 prime UTR	681	672
5 prime UTR Truncation	2	2
5 prime UTR	5412	5307
Splice acceptor	1507	1454
Splice donor	1640	1553
Splice region	14,544	14,262
Exon Synonymous	49,141	48,702
Non synonymous	33,902	33,313
Non coding exon	22,865	22,315
Non coding transcript	52	52
Exon loss	12	12
Initiator codon	7	8
Start lost	97	92
Stop gained	541	528
Stop lost	44	43
Stop retained	28	28
Transcript ablation	1	1
In frame deletion	349	330
In frame insertion	674	646
Disruptive in frame deletion	476	447
Disruptive in frame insertion	607	592
Frameshift	16,997	16,661

### Regions of homozygosity

**Fig. 2:**
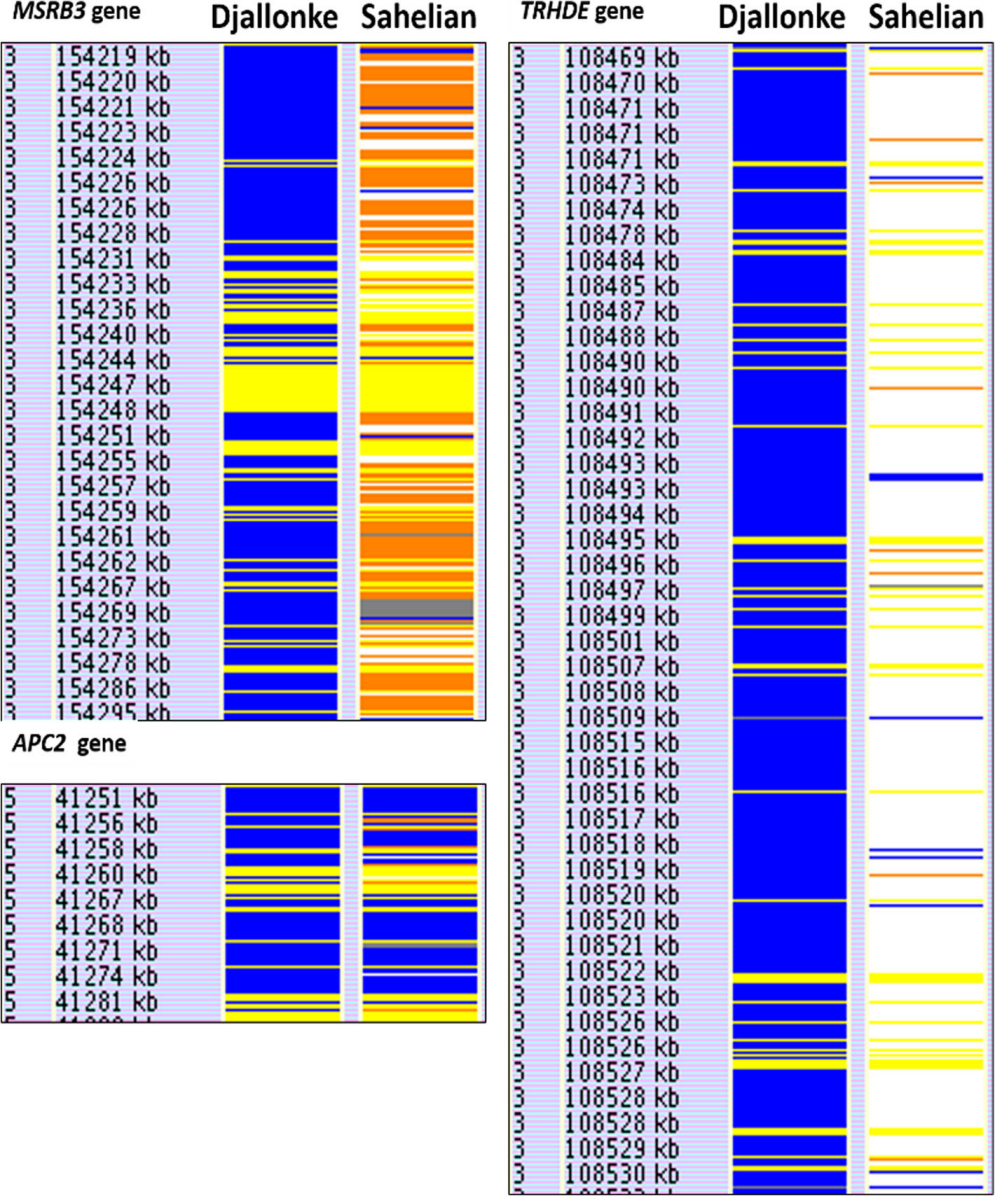
Comparison of the HomSI analysis of three genes associated with adaptive traits for Djallonke and Sahelian sheep genomes

Approximately 2.5 Gbp of autosomal chromosomal DNA was resolved into about 50,000 detection windows for each breed. HomSI analysis, identified regions having reduced heterozygosity (Fig. 2; blue) of various sizes (1 to > 100 kb) across genic and intergenic regions within both breeds. Seventy of these reduced heterozygosity regions co-localised with known candidate genes. There were also several genic and intergenic regions that showed reduced heterozygosity but did not contain any known putative candidate gene. In addition, there were regions for which the Djallonke show complete fixation for one allele (blue) and the Sahelian showing complete fixation for the alternative allele (white) e.g. *TRHDE* (Fig. 2).

### Putative regions for tolerance to trypanosomiasis

**Fig. 3:**
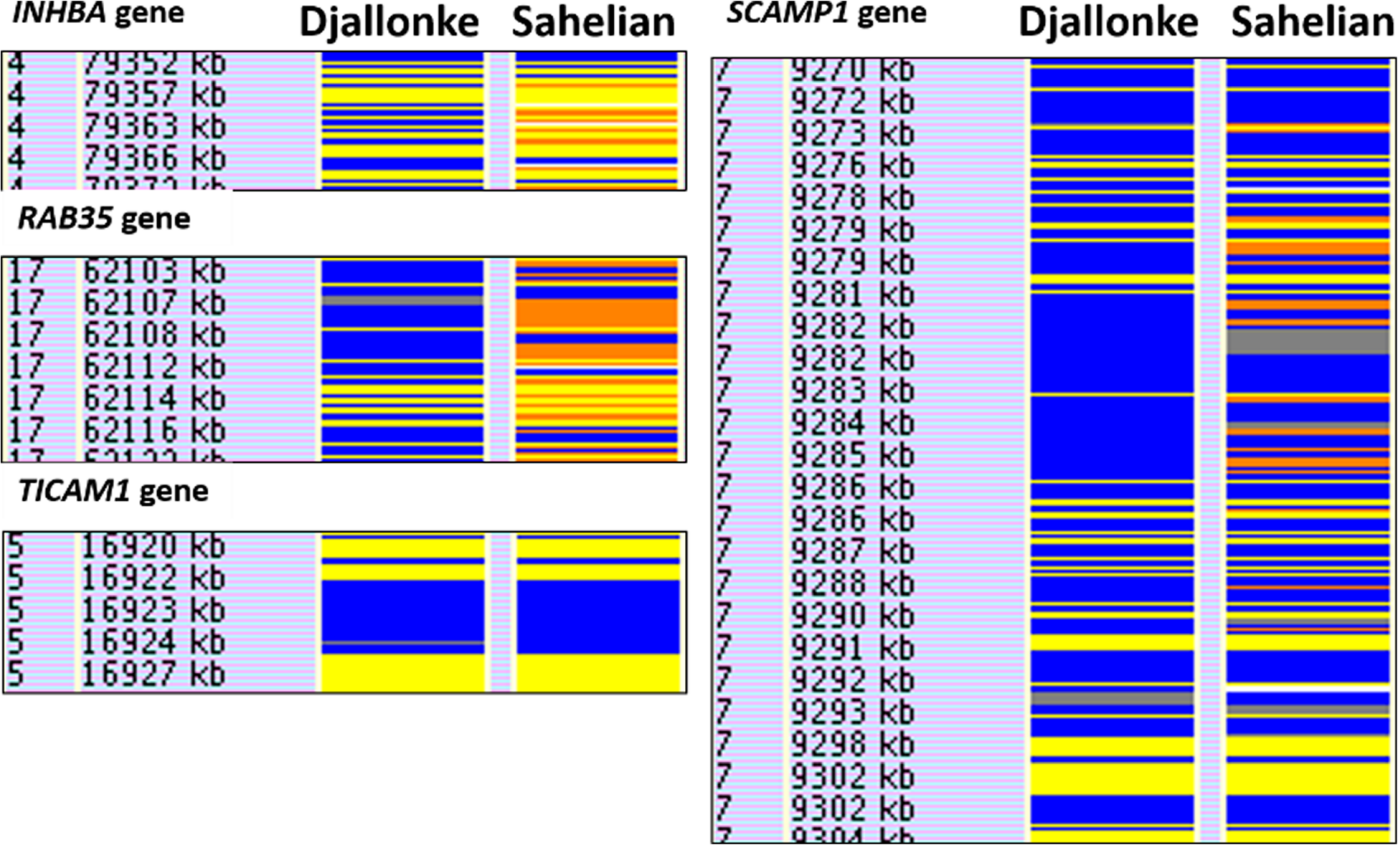
Comparison of the HomSI analysis of four Trypanotolerance associated genes for Djallonke and Sahelian sheep genomes

Eight regions of reduced heterozygosity were observed to be co-localised with previously reported trypanotolerance associated genes (Table 4) [42, 47, 48]. Six of the eight reported genes (*CTSS, ARHGAP15, INHBA, STX7, RAB35, CD19*) were co-localised with regions of reduced heterozygosity in the Djallonke genome only, and the other two (*SCAMP1, TICAM1*) were co-localised with regions of reduced heterozygosity in both Djallonke and Sahelian genomes (Fig. 3). The Djallonke sheep show longer runs of reduced heterozygosity (blue) than the Sahelian sheep at the *INHBA* and *RAB35* gene regions, but both breeds show reduced heterozygosity across approximately 2-kb (16,922 kb–16,924 kb) of the TICAM1 gene (Fig. 3). The Sahelian breed shows increased heterozygosity (orange) between 9279 kb to 9286 kb in the *SCAMP1* region. In contrast, the Djallonke shows reduced heterozygosity within the same region (blue).

**Table 4:** Trypanotolerance candidate genes co-localised with regions of reduced heterozygosity (RORH) in Djallonke and Sahelian sheep and the reported orthologs in the cattle genome

Sheep Breed	Genomic region (bp) co-localised with RORH (Oar_v3.1)	Candidate genes	Orthologous loci in Cattle UMD v 3.1
Djallonke	1:99,623,159-99,650,475	*CTSS*	3:20,024,302-20,047,228
Djallonke	2:165,154,368-165,495,812	*ARHGAP15*	2:53,065,587-53,732,838
Djallonke	4:79,355,697-79,366,930	*INHBA*	4:79,986,254-79,997,754
Both	7:9,272,393-9,359,760	*SCAMP1*	10:9,369,310-9,520,700
Both	5:16,922,626-16,924,761	*TICAM1*	7:20,547,964-20,550,264
Djallonke	8:57,581,762-57,631,158	*STX7*	9:71,381,757-71,455,585
Djallonke	17:62,105,682-62,115,444	*RAB35*	17:64,724,244-64,742,928
Djallonke	24:25,905,178-25,915,071	*CD19*	25:164,039-26,169,956

### Putative regions for resistance to Haemonchosis

**Fig. 4:**
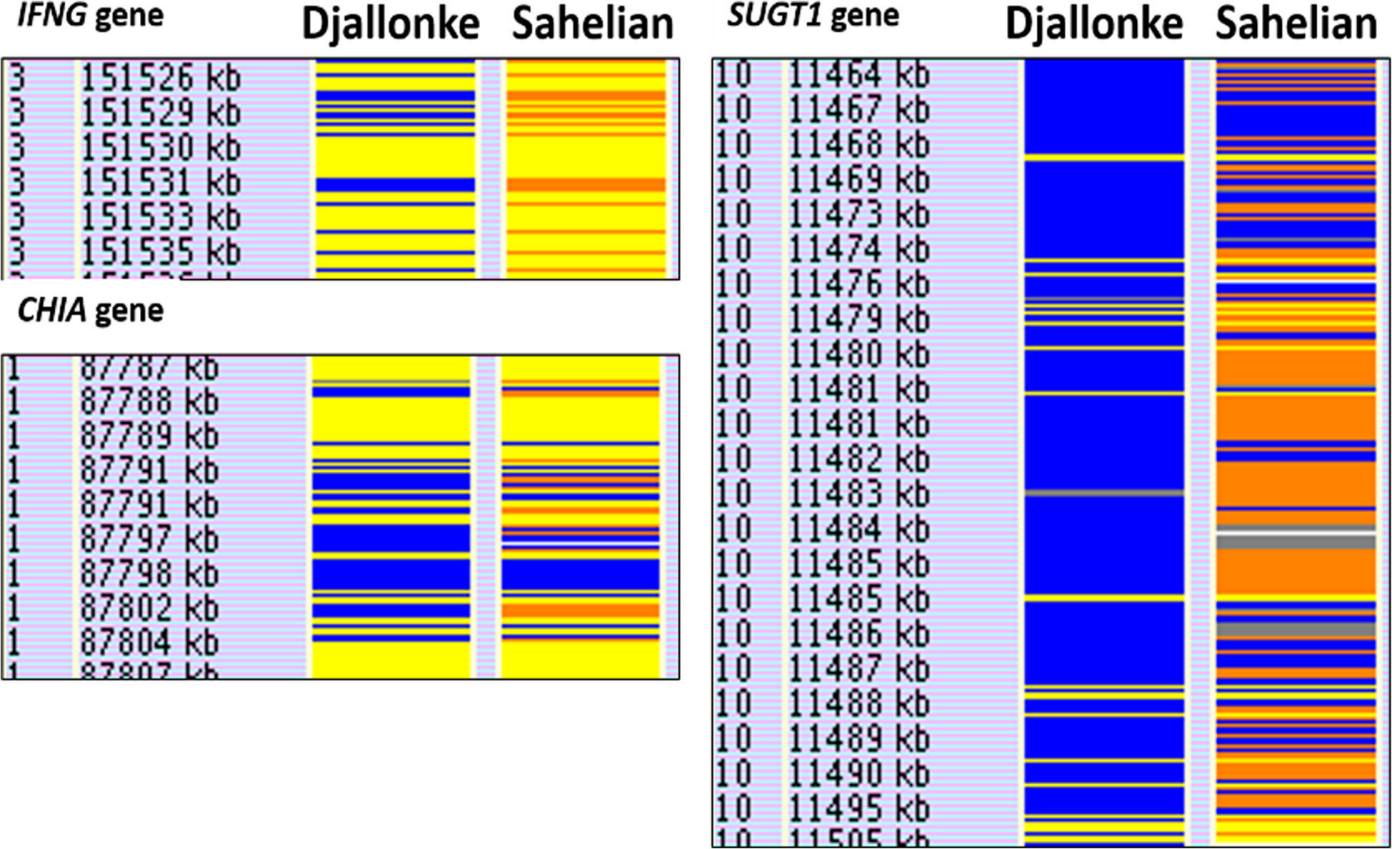
Comparison of the HomSI analysis of three Haemonchotolerance associated genes for Djallonke and Sahelian sheep genomes

There were 25 regions of reduced heterozygosity that co-localised with previously reported candidate gene for Haemonchosis resistance (Table 5). Twenty-one of the regions had reduced heterozygosity in the Djallonke sheep. The remaining four regions *(MHCII-DRB1, PIK3CD, MUC15, IL17RB*) had reduced heterozygosity in both Djallonke and Sahelian sheep. Figure 4 shows three regions associated with resistance to *Haemonchus contortus* infection: the *IFNG* gene, the *CHIA* gene, and the *SUGT1* gene. In each case, only the Djallonke sheep displayed reduced heterozygosity.

**Table 5:** Regions of reduced heterozygosity (RORH) in Djallonke and Sahelian sheep co-localised with reported candidate genes for resistance to nematodes

Sheep breed	Genomic region (bp) co-localised with RORH (Oar_v3.1)	Candidate Gene	Trait Inference	Reference
Both	20:25,400,738-25,402,966	*MHCII-DRB1*	Gastrointestinal nematodes	Schwaiger et al. (1995)
Djallonke	1:27,524,283-27,601,025	*LRP8*	*H. contortus*	Benavides et al. (2016)
Djallonke	1:87,657,990-87,674,113	*DENND2D*	*H. contortus*	McRae et al. (2014)
Djallonke	1:87,710,082-87,728,410	*CHI3L2*	*H. contortus*	McRae et al. (2014)
Djallonke	1:87,788,905-87,811,255	*CHIA*	*H. contortus*	McRae et al. (2014)
Djallonke	3:151,527,165-151,535,188	*IFNG*	Mixed intestinal parasites	Coltman et al., 2001
Djallonke	3:123,851,175-125,982,479	*ATP2B1*	*H. contortus*	Benavides et al. (2016)
Djallonke	8:62,006,022-62,039,859	*IL20RA*	*H. contortus* + others	Periasamy et al., 2014
Both	12:41,923,922-41,973,979	*PIK3CD*	*H. contortus* + others	Periasamy et al., 2014
Djallonke	12:62,163,057-62,283,591	*LAMC1*	*H. contortus*	Benavides et al. (2016)
Both	15:55,404,807-55,417,235	*MUC15*	*H. contortus*	Benavides et al. (2016)
Djallonke	17:52,168,248-52,191,479	*ABCB9*	*H. contortus*	Yang et al., 2015
Djallonke	10:11,465,154-11,505,274	*SUGT1*	*H. contortus*	Yang et al., 2015
Djallonke	14:48,062,306-48,071,138	*PAK4*	*H. contortus*	Yang et al., 2015
Djallonke	14:14,206,784-14,215,316	*FCER2*	*H. contortus*	Yang et al., 2015
Djallonke	4:44,668,137-45,205,142	*RELN*	*H. contortus*	McRae et al., 2014
Djallonke	6:89,053,717-89,061,196	*AREG*	*H. contortus*	Zhengyu et al., 2016
Djallonke	11:57,796,766-57,800,093	*SOX9*	*H. contortus*	Benavides et al., 2016
Djallonke	6:70,189,729-70,234,612	*KIT*	*H. contortus*	Zhengyu et al., 2016
Djallonke	16:66,450,575-66,482,861	*NSUN2*	*H. contortus*	McRae et al., 2014
Both	19:47,044,394-47,059,322	*IL17RB*	*H. contortus*	Zhengyu et al., 2016
Djallonke	19:55,173,564-55,175,033	*HRH1*	*H. contortus*	McRae et al., 2014
Djallonke	25:44,535,996-44,543,183	*CXCL12*	*H. contortus*	Zhengyu et al., 2016
Djallonke	19:53,290,059-53,291,096	*CXCR6*	*H. contortus*	Zhengyu et al., 2016
Djallonke	20:34,055,201-34,055,659	*UBE2N*	*H. contortus*	Benavides et al., 2016

### Putative regions for adaptation to tropical conditions

There were also genomic regions of reduced heterozygosity that were co-localised with genes known to be associated with immune responses and natural adaptation (Fig. 2). A total of 37 candidate genes fell within these reduced heterozygosity genomic regions in the Djallonke sheep, including 14 that were shared with the Sahelian sheep (Table 6). Three of the gene regions associated with adaptive selection (*MSRB3* gene, *APC2* gene, and *TRHDE* gene) are shown in Fig. 2. Differences between these genes were observed with respect to the polymorphism patterns. The Djallonke sheep have reduced heterozygosity (blue) at the region of the *MSRB3* gene whilst the Sahelian was more polymorphic (Fig. 2). Over much of the *TRDHE* gene region, the two sheep breeds were fixed for alternative alleles. Both sheep breeds, however, showed reduced heterozygosity for the same allele between coordinates 41,267,000 and 41,272,000, encompassing 7 exons (exons 16 to 22) of the *APC2* gene.

**Table 6:** Candidate genes for tropical adaptation co-localised with regions of reduced heterozygosity in Djallonke and Sahelian sheep

Sheep	Chr.	Genomic coordinates	Candidate gene	Trait Inference	Reference
DJ	1	85,955,810-86,011,841	*GNAI3*	Melanogenesis (Thermo-tolerance)	Kim et al., 2016
DJ	1	188,388,916-188,441,236	*LMLN*	Melanogenesis	Kim et al., 2016
DJ	1	42,584,598-42,656,778	*IL12RB2*	Immune functions	Roffler et al., 2016
DJ	1	168,393,395-168,624,986	*ALCAM*	Immune functions	Roffler et al., 2016
DJ	1	121,075,675-121,168,117	*SYNJ1*	Phosphatidylinositol dephosphorylation	Roffler et al., 2016
Both	2	52,423,842-52,445,175	*NPR2*	Skeletal Morphology and body size	Kijas et al., 2012
DJ	4	9,433,282-9,465,962	*KRIT1*	Regulation of endothelial cell proliferation and migration	Roffler et al., 2016
DJ	4	85,316,865-85,381,180	*TSPAN12*	Regulation of signal transduction of cell surface receptors	Roffler et al., 2016
Both	5	41,256,802-41,272,546	*APC*	Immune functions (Tumour suppressor)	Roffler et al., 2016
Both	6	36,514,210-36,556,824	*ABCG2*	Urea Metabolism (Homeostasis)	Kijas et al., 2012
Djallonke	6	94,584,400-94,605,575	*FGF5*	Regulation of fibroblast growth factor receptor	Kijas et al., 2012
Both	7	63,450,344-63,456,226	*BMP4*	Body size and development	Kim et al., 2016
DJ	3	204,447,104-204,461,390	*OLR1*	Internalization, degradation of oxidised low density lipoprotein by endothelial cells	Roffler et al., 2016
Djallonke	3	108,235,641-108,685,027	*TRHDE*	Regulation of appetite and digestion	Kim et al., 2016
Djallonke	3	154,219,234-154,397,986	*MSRB3*	Regulation of response to oxidative stress	Kijas et al., 2012
Both	3	35,907,955–36,031,445	*ALK*	Immune function (Protein phosphorylation)	Kim et al., 2016
Both	3	99,472,045-99,509,159	*IL1R1*	Immune function	Kim et al., 2016
Djallonke	9	54,817,997-54,825,977	*IL7*	Immune function	Kim et al., 2016
Both	10	36,838,524-36,858,872	*ATP12A*	Homeostasis (Potassium and Sodium)	Kim et al., 2016
Djallonke	10	40,800,056-40,821,770	*PCDH9*	Homophilic cell adhesion	Kim et al., 2016
Both	11	36,083,204-36,098,540	*PDK2*	Homeostasis	Kijas et al., 2012
Both	11	18,245,395-18,411,418	*NF1*	Homeostasis	Kijas et al., 2012
Both	13	78,815,423-78,893,076	*NFATC2*	Immune function	Kijas et al., 2012
DJ	13	666,266-1,154,524	*PLCB1*	Thermotolerance	Kim et al., 2016
DJ	15	45,551,281-45,552,222	*OR2AG1*	Response to stimulus	Kijas et al., 2012
Both	16	38,969,273-39,028,126	*PRLR*	Reproduction	Kijas et al., 2012
DJ	17	18,131,831-18,226,233	*ELF2*	Regulation of transcription	Kim et al., 2016
DJ	17	29,240,707-29,257,289	*PGRMC2*	Reproduction	Kim et al., 2016
DJ	18	19,723,286-19,802,578	*ABHD2*	Wound healing	Kijas et al., 2012
DJ	18	4,690,980-4,728,935	*ALDH1A3*	Energy, digestive Metabolism	Kim et al., 2016
Both	18	38,107,388-38,110,333	*FOXG1*	Regulation of transcription	Kijas et al., 2012
Both	19	31,583,789-31,811,540	*MITF*	Melanogenesis	Kijas et al., 2012
DJ	19	7,255,507-7,331,066	*GLB1*	Cellular metabolism	Kijas et al., 2012
DJ	19	33,852,131-34,140,194	*SUCLG2*	Cellular metabolism	Kim et al., 2016
Both	20	26,649,266-26,651,191	*HSPA1A*	Homeostasis	
Both	21	49,011,232-49,012,130	*IFITM21*	Immune functions	Roffler et al., 2016
DJ	21	42,526,284-42,531,851	*BATF2*	Immune functions	Roffler et al., 2016

## Discussion

A comparison of the genomes of Djallonke and Sahelian sheep in this study has shown that Djallonke sheep have a genetic variant every 191 base pairs while the Sahelian sheep have a genetic variant every 193 base pairs. Approximately 16% of the variants had not been previously reported in sheep. The two breeds also had similar ratios of transitions to transversions (2.5), missense to silent mutations 90.7) and insertions to deletions (0.4). These breeds also had similar proportions of SNP to indels. The distribution of variants across the autosomal chromosomes was also similar; in both breeds chromosome 11 had the lowest frequency of variants while chromosome 26 had the highest frequency.

The transition to transversion ratio obtained for Djallonke (2.47) and Sahelian (2.48) are similar to the expected values observed for other mammalian genomes: 2.26 for cattle whole genome [49], 2.13 for human intergenic SNPs [50], and 2.81 for human exonic SNPs [51]. These comparable ratios support the reliability of the sequenced datasets in this study, and they are therefore expected to contain low numbers of false positives (Type 1 errors) caused by random sequencing errors. This is further underscored by the high sequencing coverage statistics obtained for both genomes (i.e. > 97% and > 20x), which is suitable for “high-confidence” variant calling [52]. The advantage that sequencing has over medium or high-density SNP genotype datasets, is that it provides higher resolution and power for the detection of selection signatures over relatively short distances [53, 54]. For instance, the Illumina Ovine 50KSNP BeadChip and Illumina Bovine HD 800KSNP BeadChip only provide a SNP density of approximately 1 SNP for every 5 million and 3 million bases, respectively. Furthermore, the use of markers on breeds that were not included in the training set for the marker development introduces further possible ascertainment bias into the analysis.

The proportion of SNPs in the intergenic, intronic and the remaining genic regions including exons for the two genomes are similar to the proportions recorded in Korean cattle breeds [49]. The exonic regions, although containing the least number of SNPs, represent the most important subset of SNP, because they are more likely to be associated with changes in protein sequence, structure and function than intronic and intergenic SNPs [55]. In particular, population-specific, rare exonic SNPs have been shown to be the most consequential determinants of fitness traits in humans [56]. Fixed non-synonymous SNPs, which are described as SNPs for which only one allele (of a given locus) is present in a population, are of major interest in identifying breed or population specific traits [49].

The high number of novel variants identified: 2,057,096 (16.03%) in the Djallonke breed and 1,983,296 (15.67%) in the Sahelian sheep confirms that these breeds are an important genetic resource for world sheep diversity. More than 0.5 million SNPs in each of the two sheep breeds are probably novel. There were also high numbers of breed specific variants; 242,572 SNP and 82,609 indels in the Djallonke and 120,652 SNP and 37,762 indels in the Sahelian breed. These breed specific variants could facilitate the sustainable management of these breeds and aid in confronting future emerging livestock diseases as well other global challenges, such as the uncertain consequences of climate change [57]. Recent reports indicate that most of the indigenous African livestock breeds are endangered [58] and might become extinct.

The HomSI scan permitted the identification of regions of reduced heterozygosity in greater detail than other sliding window algorithms such as the “Integrated haplotype homozygosity score (iHS)” [59, 60] and “the composite of likelihood ratio (CLR)” statistics [53, 61]. Furthermore, iHS detects only “ongoing sweeps” and CLR detects only “completed sweeps” in a target genome. Additionally, selection sweeps identified using HomSI are of higher resolution in comparison to the other methods (with sliding windows of 10,000 versus 50,000 base pairs). Runs of reduced heterozygosity were identified in reported candidate gene regions and may have resulted from selective sweeps. There were 70 regions of identified to have relatively reduced heterozygosity that co-localised with previously reported candidate genes for tolerance to trypanosomiasis, resistance to haemonchosis or adaptation to tropical conditions.

Five of the eight candidate trypanotolerance genes previously reported in a peripheral blood mononuclear cell gene expression study in experimentally infected trypanotolerant Ndama cattle [48] fell within the regions of reduced heterozygosity identified in this study (*STX7, SCAMP1, RAB35, CD19, CTSS*). We identified putative selection signatures that co-localised with four of these five genes in Djallonke sheep, but the fifth candidate gene (*SCAMP1*) had similar values of heterozygosity in both Djallonke and Sahelian sheep. It is possible that Sahelian sheep may have also undergone some selection for trypanotolerance. Interestingly a sixth candidate gene, the INHBA gene, also fell within a region of reduced heterozygosity in the Djallonke. The INHBA candidate loci is the most significantly associated trypanotolerant loci reported in the Animal QTLdb to date [35]. The *INHBA* gene was identified through fine mapping analysis of four a priori identified trypanotolerant associated loci in 360 Ndama cattle under natural infection conditions [42]. The *INHBA* gene has been shown to regulate the differentiation of hematopoietic cells in mammals [62–65]. This is consistent with the hypothesised mechanisms of trypanotolerance, because the trait is strongly associated with the host’s capacity to control anaemia [4, 42, 66]. The last two trypanotolerance candidate genes, *ARHGAP15* and *TICAM1,* fell within regions of reduced heterozygosity. These two genes were previously identified in a combined transcriptomic and selective sweep analysis of infected trypanotolerant Ndama and Boran cattle [43]. These genes co-localised within regions of reduced heterozygosity in the Djallonke dataset, whereas only *TICAM1,* but not *ARHGAP15,* was co-localised with a region of reduced heterozygosity in the Sahelian dataset.

Previous studies on trypanotolerance have used a lower density of molecular markers [41, 42, 47, 48], and the confidence limits of the reported candidate loci are quite large [16]. Comparison of the Djallonke and Sahelian sheep revealed several putative selective sweeps of varying sizes (down to 2 Kilo-bases resolution). Although trypanotolerance is a complex quantitative trait and controlled by many genes, it is highly unlikely that all of the variants captured in these regions are causative variants. It is more likely that some variants are in linkage disequilibrium with the causal variants and hitch-hiked over time [67].

Gene ontology (GO) revealed that the 25 haemonchosis associated regions contain genes involved in multiple biological processes such as immune response and chemotaxis (*MHCII-DRB1, IL20RA, IL17RB, FCER2, HRH1*), response to pain and tissue homeostasis (*RELN, SOX9*), and protein coding, binding, methylation and phosphorylation (*ATP2B1, SOX9, MUC15, UBE2N, LRP8, RELN, NSUN2, LAMC1, ABCB9, PIK3CD, SUGTI, PAK4*) [32, 33]. Other functions of the identified candidate genes include calcium binding and transport (*LRP8, LAMC1*) and carbohydrate metabolism (*CHI3L2, CHIA*) [32, 33].

Six of the genes falling within regions of reduced heterozygosity identified in this study (*LRP8*, *ATP2B1*, *LAMC1*, *SOX9*, *MUC15, UBE2N)* were also associated with resistance to *H. contortus* infection in a recent GWAS study using a backcross population of Red Maasai and Dorper sheep under natural infection conditions [38]. A further six genes (*CHI3L2*, *CHIA*, *DENND2D*, *RELN*, *NSUN2*, and *HRH1)* were among the previously reported top 1% of candidate genes for resistance and susceptibility to gastrointestinal nematodes in divergent populations of Romney and Perendale sheep [37]. Two of the genes (*IL20RA*, *PIK3CD*) were associated with resistance to experimental challenge with *H. contortus* [36]. In a gene expression study of deliberately infected Chinese Hu sheep, four genes (*ABCB9*, *SUGT1*, *PAK4*, *FCER2)* were found to contribute to the key immunological responses [39]. More recently, five of the genes (*AREG*, *KIT*, *IL17RB*, *CXCL12*, *CLCR6)* were also found to be up regulated in *H. contortus* resistant Canarian hair sheep [40]. Three of the 23 genes (*IL20RA, PIK3CD, RELN*) have also been associated with resistance to other gastrointestinal nematodes such as *Trichostrongyle species, Teladorsagia circumcincta* and other *Nematodirus species* [36, 38].

A total of 37 regions with reduced heterozygosity contained genes associated with adaptive responses. Some of the genes were involved in immune functions (e.g. *IL12RB2, ALCAM*, *APC2, IL1R, 1IL7*), homeostasis (e.g. *HSPA1A, ATP12A, PDK2, NF1, ABCG2*), melanogenesis/ thermotolerance (*GNAI3, LMLN, PLB1, MITF*) and cellular and digestive metabolism (*GLB1, SUCLG2, TRHDE, OLR1*) [31, 68, 69]. These genes are plausible candidates for resistance to disease, heat tolerance, or the ability to exist on low quality diets in the harsh, hot and humid climatic conditions faced by these sheep breeds.

Twelve of the 37 low heterozygosity regions (*NPR2*, *ABCG2*, *FGF5*, *MSRB3*, *PDK2*, *NF1*, *NFATC2*, *OR2AG1*, *PRLR*, *ABHD2*, *MITF*, *GLB1*) were also reported in the top 0.1% of candidate genes identified in a previous genome-wide study for signatures of recent selection in 74 different sheep breeds selected from various regions of the world [68]. Fourteen of the 37 regions (*GNAI3*, *LMLN*, *BMP4*, *TRHDE*, *ALK*, *IL1R1*, *IL7*, *ATP12A*, *PCDH9*, *PLCB1*, *ELF2*, *PGRMC2*, *ALDH1A3*, and *SUCLG2*) were also among the genes recently reported as candidate adaptive genes in indigenous Egyptian sheep and goat breeds [31].

More recently, in a study of natural local environmental adaptation, ten regions (*IL12RBB2*, *ALCAM*, *SYNJ1*, *KRIT1*, *TSPAN12*, *APC*, *OLR1*, *IFTM21*, and *BATF2*) were among those reported as being important for adaptation in Dall sheep (*Ovis dalli dalli*) [69]. This approach combined targeted resequencing of a priori identified candidate adaptive genes of immunity and metabolism in domestic sheep (*O. aries*) and bighorn sheep (*Ovis canadensis*) to develop a panel of SNP markers [69]. As with Djallonke sheep, Dall sheep have undergone many centuries of natural selection with limited human intervention. In contrast to the tropical climatic conditions for Djallonke and Sahelian sheep, the Dall sheep breed evolved under Arctic and sub-Arctic climatic challenges, and hence the common swept regions have direct bearing to only immune functions and not climatic adaptation.

The many shared adaptive signatures of selection between the Djallonke and Sahelian sheep in this study can be attributed to common selection pressures due to their shared environment over several centuries. Historical admixture has been reported in Djallonke sheep populations in different regions of sub-Saharan Africa [70, 71]. The high number of shared variants (96%) also supports the possibility of migration between the breeds. Introgression from a breed with a high frequency of a homozygous region may reduce heterozygosity in the recipient breed.

## Conclusions

A whole genome analysis of the Djallonke and Sahelian sheep breeds identified over 1 million novel genomic variants. This large number of novel variants suggests that the two sheep breeds represent unique genetic resources, and hence are important for world sheep diversity. The considerable number of breed-specific SNPs identified in Djallonke and Sahelian sheep could aid the sustainable management of each breed. The results also appear to support previous reports of genetic regions associated to trypanotolerance, resistance to *H. contortus* infection and adaptation to a harsh tropical climate. The genomic evidence of trypanotolerance, inferred from conserved orthologues of trypanotolerant Ndama cattle, suggests evidence of similar adaptive selection response for a common disease in two different ruminant species. However, a more comprehensive genetic study in a larger dataset coupled with clinical parasitology will be required to a make any definitive statement.

## Supplementary information

**Additional file 1: ** Shows the details of Genomic relationship matrix output for this analysis for all the individual Djallonke and Sahelian sheep sampled for this study.

## Data Availability

The data has been generated from this study has been release to the European variant archive of the European Bioinformatics Institute with the accession number PRJEB15642. The data has also been shared with the International Sheep Genomics Consortium.

## Abbreviations

Animal QTLdb: Animal Quantitative Trait Loci
CLR: composite of likelihood ratio
DJ: Djallonke
GATK: Genome Analysis Tool Kit
GCTA: Genome-wide Complex Trait Analysis
GRM: Genomic relationship matrix
HomSI: Homozygosity Stretch Identifier
iHS: Integrated haplotype homozygosity score
PCA: Principal component analysis
SA: Sahelian
SNP: Single nucleotide polymorphism
UTR: Untranslated regions
