# Supplementary material for: Analysis of pooled genome sequences from Djallonke and Sahelian sheep of Ghana reveals co-localisation of regions of reduced heterozygosity with candidate genes for disease resistance and adaptation to a tropical environment
Source: BMC Genomics. 2019 Nov 7;20:816. doi: 10.1186/s12864-019-6198-8 (PMC6836352; doi:10.1186/s12864-019-6198-8)
Supplement: Supplementary file 1 — Additional file 1: Shows the details of Genomic relationship matrix output for this analysis for all the individual Djallonke and Sahelian sheep sampled for this study. [file 12864_2019_6198_MOESM1_ESM.docx]

**Table S1** GRM confirming that all ten individual animals sampled (1 to 10) for this study were unrelated

| Sample ID |  | No. of non-missing SNPs | Genetic relationship matrix |
| --- | --- | --- | --- |
| 1 | 1 | 9.30E+06 | 1.61E+00 |
| 2 | 1 | 8.37E+06 | -1.99E-01 |
| 2 | 2 | 9.64E+06 | 1.65E+00 |
| 3 | 1 | 7.48E+06 | -2.49E-01 |
| 3 | 2 | 7.72E+06 | -2.51E-01 |
| 3 | 3 | 8.52E+06 | 1.55E+00 |
| 4 | 1 | 8.04E+06 | -2.05E-01 |
| 4 | 2 | 8.28E+06 | -2.23E-01 |
| 4 | 3 | 7.41E+06 | -2.51E-01 |
| 4 | 4 | 9.19E+06 | 1.60E+00 |
| 5 | 1 | 7.87E+06 | -2.35E-01 |
| 5 | 2 | 8.12E+06 | -2.41E-01 |
| 5 | 3 | 7.28E+06 | -1.31E-01 |
| 5 | 4 | 7.78E+06 | -2.23E-01 |
| 5 | 5 | 8.99E+06 | 1.54E+00 |
| 6 | 1 | 5.27E+06 | -2.71E-01 |
| 6 | 2 | 5.39E+06 | -2.79E-01 |
| 6 | 3 | 4.95E+06 | -2.72E-01 |
| 6 | 4 | 5.22E+06 | -2.76E-01 |
| 6 | 5 | 5.14E+06 | -2.83E-01 |
| 6 | 6 | 7.79E+06 | 1.68E+00 |
| 7 | 1 | 5.26E+06 | -3.23E-01 |
| 7 | 2 | 5.38E+06 | -3.16E-01 |
| 7 | 3 | 4.94E+06 | -3.10E-01 |
| 7 | 4 | 5.21E+06 | -3.17E-01 |
| 7 | 5 | 5.13E+06 | -3.14E-01 |
| 7 | 6 | 6.86E+06 | -2.55E-01 |
| 7 | 7 | 7.75E+06 | 1.53E+00 |
| 8 | 1 | 4.20E+06 | -3.58E-01 |
| 8 | 2 | 4.25E+06 | -4.07E-01 |
| 8 | 3 | 3.98E+06 | -2.59E-01 |
| 8 | 4 | 4.17E+06 | -3.31E-01 |
| 8 | 5 | 4.09E+06 | -3.14E-01 |
| 8 | 6 | 5.17E+06 | -2.72E-01 |
| 8 | 7 | 5.13E+06 | -1.57E-01 |
| 8 | 8 | 5.71E+06 | 1.68E+00 |
| 9 | 1 | 4.79E+06 | -3.44E-01 |
| 9 | 2 | 4.88E+06 | -3.62E-01 |
| 9 | 3 | 4.52E+06 | -2.95E-01 |
| 9 | 4 | 4.75E+06 | -3.32E-01 |
| 9 | 5 | 4.67E+06 | -3.22E-01 |
| 9 | 6 | 6.10E+06 | -1.75E-01 |
| 9 | 7 | 6.07E+06 | -7.65E-02 |
| 9 | 8 | 4.72E+06 | -4.83E-02 |
| 9 | 9 | 6.83E+06 | 1.52E+00 |
| 10 | 1 | 4.91E+06 | -3.35E-01 |
| 10 | 2 | 5.01E+06 | -3.47E-01 |
| 10 | 3 | 4.63E+06 | -2.92E-01 |
| 10 | 4 | 4.86E+06 | -3.20E-01 |
| 10 | 5 | 4.80E+06 | -3.11E-01 |
| 10 | 6 | 6.31E+06 | -2.68E-01 |
| 10 | 7 | 6.29E+06 | -1.09E-01 |
| 10 | 8 | 4.80E+06 | -4.30E-02 |
| 10 | 9 | 5.63E+06 | -1.40E-01 |
| 10 | 10 | 7.09E+06 | 1.57E+00 |
